# Double aortic arch: a rare cause of stridor in infants

**DOI:** 10.1093/jscr/rjab567

**Published:** 2021-12-24

**Authors:** Youssef Aladham, Quentin Bonduelle, John Yaro, Omar Ahmed

**Affiliations:** Department of Otolaryngology, University Hospitals of Derby and Burton, Derby, UK; Department of Otolaryngology, East Kent Hospitals University NHS Foundation Trust, Ashford, UK; Department of Otolaryngology, University Hospitals of Derby and Burton, Derby, UK; Department of Otolaryngology, East Kent Hospitals University NHS Foundation Trust, Ashford, UK; Department of Otolaryngology, University Hospitals of Derby and Burton, Derby, UK; Department of Otolaryngology, East Kent Hospitals University NHS Foundation Trust, Ashford, UK; Department of Otolaryngology, University Hospitals of Derby and Burton, Derby, UK; Department of Otolaryngology, East Kent Hospitals University NHS Foundation Trust, Ashford, UK

## Abstract

Double aortic arch is the most common vascular ring anomaly. It usually presents with symptoms related to tracheal and oesophageal compression. The constricting vascular ring may lead to stridor in infants and young children, which could be mistaken for upper respiratory tract infections or foreign body aspiration. It is therefore prudent to have a high index of suspicion when evaluating cases of paediatric stridor. Contrast-enhanced computed tomography and cardiac magnetic resonance imaging are the diagnostic modalities of choice to investigate vascular rings. We report a case of a stridulous infant with a double aortic arch.

## INTRODUCTION

Stridor in infants is a common paediatric ear, nose, throat presentation. Foreign body aspiration and upper respiratory tract infections are by far the most common causes of stridor in this age group. A paediatric otolaryngologist should always bear in mind other less common causes of airway compromise. Double aortic arch is the most common type of vascular ring anomalies. It encircles the trachea leading to airway compression and stridor. The age of presentation varies from the early neonatal period to adulthood [1]. We report an infant with double aortic arch, presenting with stridor mimicking foreign body aspiration.

## CASE REPORT

A 1-month-old infant presented with progressive stridor that was noticed by the parents to have got worse for 2 months. They did not recall a specific chocking event or a recent respiratory tract infection. The infant was born full-term and had no significant medical or surgical history.

A moderate degree of biphasic stridor, with both suprasternal and intercostal recessions, was noted on examination. Oxygen saturations on room air were 94% and the infant was afebrile. Chest auscultation revealed bilaterally reduced, yet equal, air entry with transmitted stridor. No lung crepitations were appreciated.

**Figure 1:**
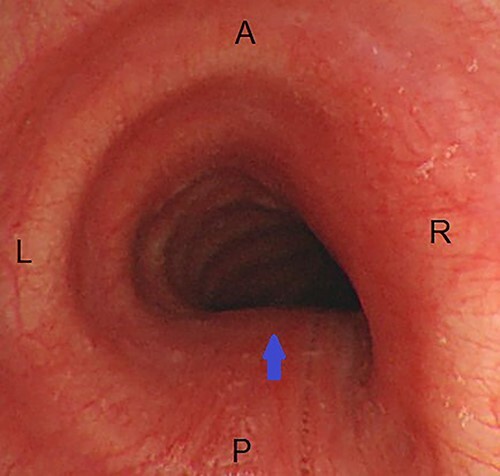
Endoscopic view of the trachea during tele-bronchoscopy; there is circumferential narrowing of the lumen, more prominent on the posterior and right sides (arrow); A, anterior; P, posterior; R, right; L, left.

A chest X-ray was normal. Suspicion of unwitnessed foreign body aspiration arose, and an elective diagnostic bronchoscopy was arranged. Bronchoscopy revealed no foreign body throughout the tracheobronchial tree, but evidence of pulsatile circumferential constriction of the lower trachea was noted, with tendency of the tracheal wall to collapse upon withdrawal of the bronchoscope (Fig. 1).

**Figure 2:**
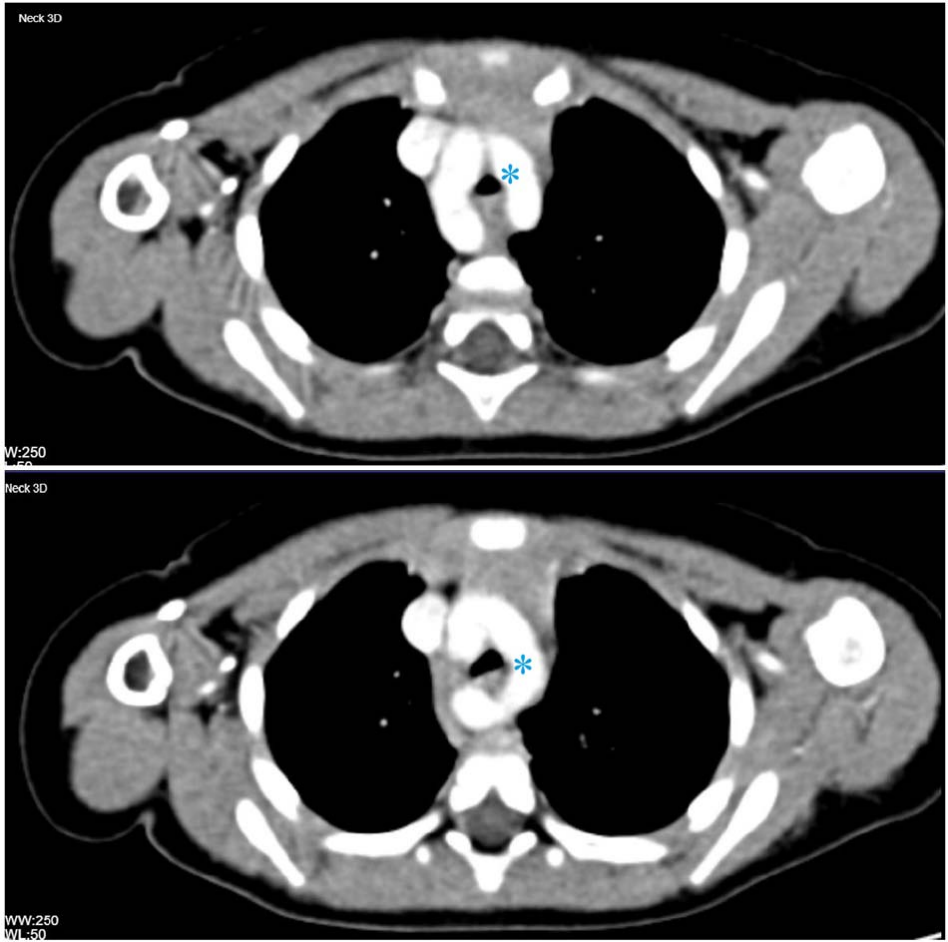
Contrast-enhanced CT of the chest in two consecutive axial cuts showing duplication of the aortic arch encircling the thoracic trachea and oesophagus (asterisk).

Post-operatively, a contrast-enhanced computed tomography (CT) of the neck and chest was obtained and a vascular ring, consisting of a double aortic arch, was found compressing and narrowing the trachea (Fig. 2).

A complete systemic work-up including echocardiography and renal ultrasound did not reveal any associated anomalies. The patient was then referred to the cardiothoracic surgery and underwent surgical repair with resection of the right arch. Further follow-up after 6 months showed resolution of the symptoms, and active observation was continued.

## DISCUSSION

Vascular ring malformations are relatively rare, and double aortic arch is their most common type. The aortic arch normally develops from the left fourth arch, while the right fourth arch becomes the right innominate artery. Double aortic arch results from failed regression of the right fourth arch during embryonic development, resulting in the formation of a vascular ring from the splitting of the ascending aorta into two limbs that pass on either side of the trachea and oesophagus, encircling and compressing them. They then rejoin as a single descending aorta [2].

The prevalence of vascular rings is estimated to be ~1% of which 55% are double aortic arch. There is no sex or race predilection. An associated cardiac anomaly is prevalent in up to 12.6%, including ventricular septal defect and Fallot’s tetralogy. It is sometimes associated with chromosome 22q11 deletion and trisomy 21 in up to 20% of the cases [3].

Double aortic arch is most diagnosed in childhood due to symptoms related to oesophageal and/or tracheal compression and obstruction. Respiratory symptoms are more common in infancy and early childhood, while adult patients tend to present more with dysphagia rather than stridor [2].

Contrast-enhanced CT and cardiac magnetic resonance imaging are the diagnostic modalities of choice for evaluating suspected vascular rings. They allow delineation of the anomaly, its location and branching pattern and arch dominance as well as determination of the severity of airway and oesophageal compression. Three-dimensional reconstruction helps to plan surgical intervention. In older patients with dysphagia, a barium swallow may show bilateral notching of the oesophagus [4].

Early surgical intervention, by resecting the minor arch freeing the trachea and oesophagus, is the only curative choice for patients with compressive symptoms. It is also of utmost importance to prevent long-term complications, such as tracheomalacia or aorto-oesophageal fistula [5].

**Learning Points**

- A paediatric otolaryngologist must have a high index of suspicion in order to diagnose vascular ring anomalies in infants and young children presenting with otherwise-unexplained stridor.
- Double aortic arch should be included in the clinical differential diagnosis of laryngo-tracheomalacia and neglected foreign body aspiration.
